# Correlations Between Inflammatory Biomarkers in Tuberculosis-Associated Obstructive Pulmonary Disease Patients With Anxiety and Depression

**DOI:** 10.7759/cureus.22742

**Published:** 2022-03-01

**Authors:** Srikanth Katare, Ajit Harsha

**Affiliations:** 1 Respiratory Medicine, Vydehi Institute of Medical Sciences and Research Centre, Bengaluru, IND

**Keywords:** hamilton anxiety rating scale (ham-a), hamilton depression rating scale (ham-d), tb -tuberculosis, combined gold copd assessment group, depression, anxiety, bio-markers, topd- tuberculosis associated obstructive pulmonary disease, tuberculosis associated copd

## Abstract

Introduction

Tuberculosis-associated obstructive pulmonary disease (TOPD), anxiety, and depression are significant public health problems worldwide and their prevalence is common. These diseases interfere with physical, psychosocial, and economic well-being, resulting in unemployment, prolonged hospitalization, abstinence from working, and isolation.

Subjects and methods

This is a single-center, cross-sectional cohort, observational study conducted in a tertiary care hospital over six years to understand spirometry, laboratory profiles, as well as the impact on overall health, daily life, and perceived well-being in patients with TOPD.

Result

The sample size of the study was 73 patients. A total of 43 (58.5%) patients had depression with an average St. George's Respiratory Questionnaire for chronic obstructive pulmonary disease (SGRQ-C) score of 67.5, and 16 (21.9%) patients had anxiety with an average SGRQ-C score of 78.9. In the patients who scored higher on the Hamilton Depression Rating Scale (HAM-D), there was a significant correlation between Hamilton Anxiety Rating Scale (HAM-A) and HAM-D scores, as well as C-reactive protein (CRP) levels and WBC counts. In 16 (21.9%) of the patients with moderate to severe anxiety, there was a statistically significant negative correlation between higher HAM-A scores and lower WBC counts. Anxiety, depression, CRP level, WBC count, and serum fibrinogen did not show a significant correlation with the Global Initiative for Chronic Obstructive Lung Disease (GOLD) groups-based assessment of TOPD severity. A high serum fibrinogen level did not correlate with a high HAM-D score, nor did a high CRP level correlate with a high HAM-A score.

Conclusion

Psychiatric comorbidities like depression are associated with increased inflammation in chronic diseases like TOPD, but no definitive biomarker has been identified and further studies are required to identify suitable biomarkers.

## Introduction

A significant sequela of pulmonary tuberculosis (TB) is irreversible airway obstruction, contributing to the development of chronic obstructive pulmonary disease (COPD). Globally, India has the highest burden of TB. Extensive fibrosis, cavitation, bulla formation, and bronchiectasis have been implicated in the development of COPD among these patients.

The high prevalence, morbidity, and mortality of COPD are major public health problems worldwide and COPD-related deaths are projected to rise to the third position within a decade. This has significant economic implications for healthcare providers, despite the availability of effective treatments and comprehensive guidelines [1,2].

Global efforts to diagnose, prevent, and treat COPD with help from the Global Initiative for Chronic Obstructive Lung Disease (GOLD) strategy document and the European Respiratory Society and American Thoracic Society have identified key pathological risk factors and proposed methods to reduce COPD-associated morbidity and mortality are being carried out [2], but TB-associated obstructive pulmonary disease (TOPD) management has received very scant attention to date.

Throughout history, TB with its sequelae and complications has always interfered with physical, psychosocial, and economic well-being, resulting in prolonged hospitalization, unemployment, abstinence from work, isolation, and hopelessness.

TB affected almost 10 million people in 2019. About 5.6 million men, 3.3 million women, and 1.2 million children were affected [3]. In 2020, Southeast Asia accounted for 43% of all TB cases, while India has accounted for 26% [4].

COPD cases have increased dramatically in India over the last three decades. They have increased from 28.1 million cases in 1990 to 55.3 million in 2016, leading to an increased prevalence from 3.3% to 4.2%. As a result, chronic respiratory diseases are disproportionately burdensome in India, causing high morbidity and mortality, particularly in the states where the epidemiological transition is relatively low [5].

A significant part of the COPD epidemic can be attributed to industrialization and the rampant spread of TB. The interaction between risk factors causing COPD and TB is both additive and synergistic [6,7]. In the PLATINO study, it was found that 30.7% of TB patients had airflow obstruction and 4.1 times greater risk of airflow obstruction among males. In conclusion, TB history is an independent risk factor for developing COPD. TOPD was described by Allwood et al. [8,9].

Anxiety and depression are important public health problems worldwide and the disease is often associated with COPD but there are no studies in association with TOPD that are of particular and increasing relevance in a polymorbid society, especially in the elderly, and the burden of the disease is expected to increase.

According to the World Health Organization (WHO), depression is among the most prevalent mental disorders in the world. The burden of depression affects more than 5% of all adults globally and is one of the major causes of disability. Depression can also lead to suicide. There are currently effective treatments for mild, moderate, and severe depression [10].

Depression and anxiety symptoms under-recognized and untreated can negatively impact physical functioning and social interaction, increasing fatigue and healthcare utilization in patients with COPD. Signs and symptoms of depression and anxiety are often overlooked in TOPD patients, making them difficult to identify and treat. There are numerous studies reporting that psychiatric morbidities such as anxiety and depression are common among patients with TB and COPD [11-13].

The association between anxiety and depression and systemic inflammation in TB and COPD (especially TOPD) patients remains unclear [14-16]. Since the prevalence of both diseases like COPD and pulmonary TB and their associated comorbidities is high among developing countries like India, this relationship would pose a significant burden on the healthcare infrastructure. Lack of literature has led to the underestimation of anxiety and depression in the Indian population with TOPD. The management of these conditions requires an in-depth evaluation of all the interrelated diseases.

Although it is becoming increasingly clear that anxiety and depression are common among chronic lung disease patients secondary to pulmonary TB, the use of instruments specifically designed to detect these symptoms is not widespread, and registries, including National Tuberculosis Elimination Program (NTEP), only capture the number of TB deaths and the number of TB-related morbidities during treatment. Therefore, patients are not followed up beyond the anti-tubercular treatment course.

Inflammation has been implicated in the pathophysiology of COPD, post-tubercular obstructive pulmonary disease, anxiety, and depression, and may influence their optimal management. Currently, there are no studies indicating definitive inflammatory biomarkers that can be used to analyze the above conditions.

In our study, we evaluated the prevalence and influence of inflammatory biomarkers in patients with depression and anxiety among TOPD patients in a tertiary care hospital in Bengaluru.

## Materials and methods

The study was conducted as a single-center study in a 1600-bed multispecialty tertiary care hospital in Karnataka, India between 2014 and 2021 after being approved by the institutional ethics committee (VIMS & RC /IEC/102/2013-2014). The current study is a cross-sectional cohort, observational study that was conducted to determine the severity of TOPD including spirometry, inflammatory biomarkers, as well as the impact on overall health, daily life, and perceived well-being in patients with TOPD.

During the course of the study period, 22,530 patients visited the outpatient department of respiratory medicine with COPD. Out of the 3,120 patients diagnosed with TOPD, 129 patients had a history of psychiatric disorders, 332 patients had a family history of psychiatric disorders, 1,036 patients had an active secondary respiratory bacterial infection, 787 patients had chronic diseases of other systems, 446 were using substances of abuse, and out of the remaining 390 patients, 73 consented to participate in the study. Well-documented cured pulmonary TB patients with no acute history of exacerbation of obstructive airway disease and having clinical features of COPD and spirometry value as per GOLD guidelines were included in the study.

All patients with chronic pulmonary diseases, sputum-positive TB, clinically suspected active pulmonary TB, active secondary respiratory bacterial infection, chronic disease of other systems, previously diagnosed psychiatric illnesses, family history of psychiatric illness, and substance abuse were excluded. The study is reported in accordance with the Strengthening the Reporting of Observational Studies in Epidemiology (STROBE) statement.

Statistical analysis was performed using Jamovi 2.0. Hamilton Anxiety Rating Scale (HAM-A), Hamilton Depression Rating Scale (HAM-D), age groups, and TOPD according to GOLD category were analyzed with Pearson's chi-squared test and linear model ANOVA. Mann-Whitney test was used to determine the significant difference between C-reactive protein (CRP) categories, white blood cells (WBC) categories, and St. George's Respiratory Questionnaire-COPD (SGRQ-C) scores, and it was expressed in terms of mean, SD, median, and interquartile range. A p-value of 0.001 was considered statistically significant.

Classification of severity of airflow limitation in TOPD patients according to the COPD GOLD guidelines (forced expiratory volume in one second (FEV1)/forced vital capacity (FVC) < 0.70) is shown in Table 1.

**Table 1:** Classification of airflow limitation severity in COPD (based on post-bronchodilator FEV1). In patients with FEV_1_/FVC < 0.70. GOLD, Global Initiative for Chronic Obstructive Lung Disease; COPD, chronic obstructive pulmonary disease; FEV_1_, forced expiratory volume in one second; FVC, forced vital capacity.

Grade	Severity	FEV_1_ of predicted
GOLD 1	Mild	FEV_1_ ≥ 80% predicted
GOLD 2	Moderate	50% ≤ FEV_1_ < 80% predicted
GOLD 3	Severe	30% ≤ FEV_1_ < 50% predicted
GOLD 4	Very severe	FEV_1_ < 30% predicted

The classification of TOPD patients is divided into GOLD ABCD groups depending on their symptoms and past exacerbation events. Table 2 illustrates the evaluation strategy.

**Table 2:** Combined COPD (ABCD) assessment groups (GOLD 2022). COPD, chronic obstructive pulmonary disease; CAT, COPD Assessment Test; mMRC, Modified Medical Research Council Questionnaire; GOLD, Global Initiative for Chronic Obstructive Lung Disease.

Exacerbation history	Groups	
Moderate or severe exacerbation history ≥2 or ≥1, leading to hospital admission	Group C	Group D
Moderate or severe exacerbation history 0 or 1, not leading to hospital admission	Group A	Group B
	mMRC = 0-1 and CAT < 10	mMRC ≥ 2 and CAT ≥ 10
	Symptom	

As described in Table 3, the HAM-A is used to measure the severity of anxiety symptoms among TOPD patients. It contains 14 items and their severity is graded as shown below.

**Table 3:** Hamilton Anxiety Rating Scale (HAM-A).

Anxiety severity	Score
Mild severity	<17
Moderate severity	18-24
Severe severity	25-30

HAM-D is used to assess the severity of depressive symptoms in TOPD patients. The scale contains 17 items, and their severity was rated in accordance with Table 4.

**Table 4:** Hamilton Depression Rating Scale (HAM-D).

Depression severity	Score
Normal	0-7
Mild depression	8-13
Moderate depression	14-18
Severe depression	19-22
Very severe depression	≥23

SGRQ-C scores range from 0 to 100, with higher scores indicating more limitations. The biomarkers studied were as follows: CRP less than 1.0 mg/dL, serum fibrinogen of 200 to 400 mg/dl, and total leukocyte count of 4.0 to 11.0 × 10^9^/L.

The COPD Assessment Test (CAT) is a patient-completed questionnaire assessing the impact of COPD (cough, sputum, dyspnea, and chest tightness) on health status. It includes eight questions on a scale from 1 to 5 and is scored 0-40. A higher score indicates a more severe impact of COPD on the patient. A target score does not represent the best outcome that may be achieved.

## Results

**Figure 1:**
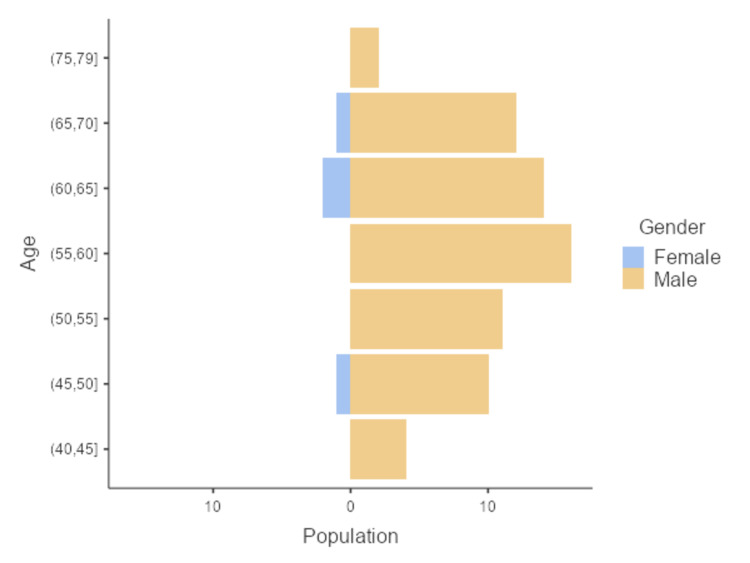
Age pyramid - distribution of the population by age group and gender.

The study involved 73 TOPD patients, of whom 69 were males and four were females. Most of the patients were between 55 and 65 years old (Figure 1). Patients ranging in age from 41 to 79 years old were interviewed; the mean age and standard deviation were 58.8 ± 8.23 years.

The TOPD patients (N = 73) were distributed and classified into the GOLD groups. Ten patients belonged to the GOLD group A, 31 patients belonged to the GOLD group B, one patient belonged to the GOLD group C, and 31 patients belonged to the GOLD group D. SGRQ-C scores and GOLD groups-based assessments of TOPD severity were having significant correlation (p < 0.001). Of the 73 TOPD patients, 43 (58.5%) had some form of depression and 16 (21.9%) had moderate to severe anxiety, as shown in Table 5.

**Table 5:** Correlation of GOLD ABCD groups with anxiety scores, depression scores, inflammatory biomarkers, and SGRQ-C scores. P < 0.001 was considered as significant. ^1^ Pearson's chi-squared test. ^2^ Linear model ANOVA. GOLD, Global Initiative for Chronic Obstructive Lung Disease; HAM-D, Hamilton Depression Rating Scale; HAM-A, Hamilton Anxiety Rating Scale; CRP, C-reactive protein; SGRQ-C, St. George's Respiratory Questionnaire for chronic obstructive pulmonary disease.

	GOLD group A (N = 10)	GOLD group B (N = 31)	GOLD group C (N = 1)	GOLD group D (N = 31)	Total (N = 73)	P-value
HAM-D score						0.251^1^
Normal	8.0 (80.0%)	14.0 (45.2%)	1.0 (100.0%)	7.0 (22.6%)	30.0 (41.1%)	
Mild depression	2.0 (20.0%)	8.0 (25.8%)	0.0 (0.0%)	9.0 (29.0%)	19.0 (26.0%)	
Moderate depression	0.0 (0.0%)	3.0 (9.7%)	0.0 (0.0%)	5.0 (16.1%)	8.0 (11.0%)	
Severe depression	0.0 (0.0%)	1.0 (3.2%)	0.0 (0.0%)	4.0 (12.9%)	5.0 (6.8%)	
Very severe depression	0.0 (0.0%)	5.0 (16.1%)	0.0 (0.0%)	6.0 (19.4%)	11.0 (15.1%)	
HAM-A score						0.239^1^
Very low anxiety	10.0 (100.0%)	26.0 (83.9%)	1.0 (100.0%)	20.0 (64.5%)	57.0 (78.1%)	
Moderate anxiety	0.0 (0.0%)	1.0 (3.2%)	0.0 (0.0%)	5.0 (16.1%)	6.0 (8.2%)	
Severe anxiety	0.0 (0.0%)	4.0 (12.9%)	0.0 (0.0%)	6.0 (19.4%)	10.0 (13.7%)	
CRP level						0.089^1^
≤1 mg/dl	9.0 (90.0%)	24.0 (77.4%)	1.0 (100.0%)	17.0 (54.8%)	51.0 (69.9%)	
>1 mg/dl	1.0 (10.0%)	7.0 (22.6%)	0.0 (0.0%)	14.0 (45.2%)	22.0 (30.1%)	
WBC/total leukocyte						0.367^1^
≤11,000 cells/cumm	10.0 (100.0%)	28.0 (90.3%)	1.0 (100.0%)	25.0 (80.6%)	64.0 (87.7%)	
>11,000 cells/cumm	0.0 (0.0%)	3.0 (9.7%)	0.0 (0.0%)	6.0 (19.4%)	9.0 (12.3%)	
Serum fibrinogen level						0.623^1^
≤200 mg/dl	0.0 (0.0%)	3.0 (9.7%)	0.0 (0.0%)	1.0 (3.2%)	4.0 (5.5%)	
200-400 mg/dl	5.0 (50.0%)	18.0 (58.1%)	1.0 (100.0%)	17.0 (54.8%)	41.0 (56.2%)	
400-600 mg/dl	2.0 (20.0%)	8.0 (25.8%)	0.0 (0.0%)	10.0 (32.3%)	20.0 (27.4%)	
≥600 mg/dl	3.0 (30.0%)	2.0 (6.5%)	0.0 (0.0%)	3.0 (9.7%)	8.0 (11.0%)	
SGRQ-C score						<0.001^2^
Mean (SD)	11.5 (12.4)	40.2 (18.8)	86.1 (NA)	75.0 (20.6)	51.7 (29.6)	
Range	0.9-35.3	0.9-72.1	86.1-86.1	18.4-98.3	0.9-98.3	

There was a significant correlation between scores on the HAM-D and scores on the HAM-A in addition to WBC counts in the patients who scored higher on the HAM-D, as shown in Table 6.

**Table 6:** Correlation of depression scores with anxiety scores, inflammatory biomarkers, and SGRQ-C scores. P < 0.001 was considered as significant. ^1^ Pearson's chi-squared test. ^2^ Linear model ANOVA. HAM-A, Hamilton Anxiety Rating Scale; CRP, C-reactive protein; SGRQ-C, St. George's Respiratory Questionnaire for chronic obstructive pulmonary disease.

	Normal (N = 30)	Mild depression (N = 19)	Moderate depression (N = 8)	Severe depression (N = 5)	Very severe depression (N = 11)	Total (N = 73)	P-value
HAM-A score							<0.001^1^
Very low anxiety	30.0 (100.0%)	18.0 (94.7%)	3.0 (37.5%)	3.0 (60.0%)	3.0 (27.3%)	57.0 (78.1%)	
Moderate anxiety	0.0 (0.0%)	1.0 (5.3%)	2.0 (25.0%)	1.0 (20.0%)	2.0 (18.2%)	6.0 (8.2%)	
Severe anxiety	0.0 (0.0%)	0.0 (0.0%)	3.0 (37.5%)	1.0 (20.0%)	6.0 (54.5%)	10.0 (13.7%)	
CRP level							0.016^1^
<1 mg/dl	25.0 (83.3%)	13.0 (68.4%)	4.0 (50.0%)	5.0 (100.0%)	4.0 (36.4%)	51.0 (69.9%)	
>1 mg/dl	5.0 (16.7%)	6.0 (31.6%)	4.0 (50.0%)	0.0 (0.0%)	7.0 (63.6%)	22.0 (30.1%)	
WBC/total leukocyte							<0.001^1^
<11,000 cells/cumm	30.0 (100.0%)	18.0 (94.7%)	7.0 (87.5%)	5.0 (100.0%)	4.0 (36.4%)	64.0 (87.7%)	
>11,000 cells/cumm	0.0 (0.0%)	1.0 (5.3%)	1.0 (12.5%)	0.0 (0.0%)	7.0 (63.6%)	9.0 (12.3%)	
Serum fibrinogen level							0.595^1^
<200 mg/dl	2.0 (6.7%)	2.0 (10.5%)	0.0 (0.0%)	0.0 (0.0%)	0.0 (0.0%)	4.0 (5.5%)	
200-400 mg/dl	16.0 (53.3%)	10.0 (52.6%)	4.0 (50.0%)	3.0 (60.0%)	8.0 (72.7%)	41.0 (56.2%)	
400-600 mg/dl	8.0 (26.7%)	3.0 (15.8%)	4.0 (50.0%)	2.0 (40.0%)	3.0 (27.3%)	20.0 (27.4%)	
>600 mg/dl	4.0 (13.3%)	4.0 (21.1%)	0.0 (0.0%)	0.0 (0.0%)	0.0 (0.0%)	8.0 (11.0%)	
SGRQ-C score							<0.001^2^
Mean (SD)	34.4 (27.8)	54.2 (21.3)	72.0 (26.2)	74.9 (34.5)	69.1 (21.1)	51.7 (29.6)	
Range	0.9-93.6	6.8-95.5	17.7-93.1	17.5-98.3	30.1-91.9	0.9-98.3	

A statistically significant negative correlation between higher HAM-A scores and WBC counts was noted. As shown in Table 7, among the 16 (21.9%) patients with moderate to severe anxiety, 10 patients had WBC counts below <11,000 cells/cumm and six patients had counts above >11,000 cells/cumm.

**Table 7:** Correlation of anxiety scores with inflammatory biomarkers and SGRQ-C scores. P < 0.001 was considered as significant. ^1^ Pearson's chi-squared test. ^2^ Linear model ANOVA. CRP, C-reactive protein; SGRQ-C, St. George's Respiratory Questionnaire for chronic obstructive pulmonary disease.

	Very low anxiety (N = 57)	Moderate anxiety (N = 6)	Severe anxiety (N = 10)	Total (N = 73)	P-value
CRP level					0.078^1^
<1 mg/dl	43.0 (75.4%)	2.0 (33.3%)	6.0 (60.0%)	51.0 (69.9%)	
>1 mg/dl	14.0 (24.6%)	4.0 (66.7%)	4.0 (40.0%)	22.0 (30.1%)	
WBC/total leukocyte					0.001^1^
<11,000 cells/cumm	54.0 (94.7%)	3.0 (50.0%)	7.0 (70.0%)	64.0 (87.7%)	
>11,000 cells/cumm	3.0 (5.3%)	3.0 (50.0%)	3.0 (30.0%)	9.0 (12.3%)	
Serum fibrinogen level					0.763^1^
<200 mg/dl	4.0 (7.0%)	0.0 (0.0%)	0.0 (0.0%)	4.0 (5.5%)	
200-400 mg/dl	30.0 (52.6%)	4.0 (66.7%)	7.0 (70.0%)	41.0 (56.2%)	
400-600 mg/dl	16.0 (28.1%)	1.0 (16.7%)	3.0 (30.0%)	20.0 (27.4%)	
>600 mg/dl	7.0 (12.3%)	1.0 (16.7%)	0.0 (0.0%)	8.0 (11.0%)	
SGRQ-C score					<0.001^2^
Mean (SD)	44.5 (27.9)	85.2 (19.7)	72.6 (19.2)	51.7 (29.6)	
Range	0.9-97.6	45.7-98.3	30.1-92.2	0.9-98.3	

**Figure 2:**
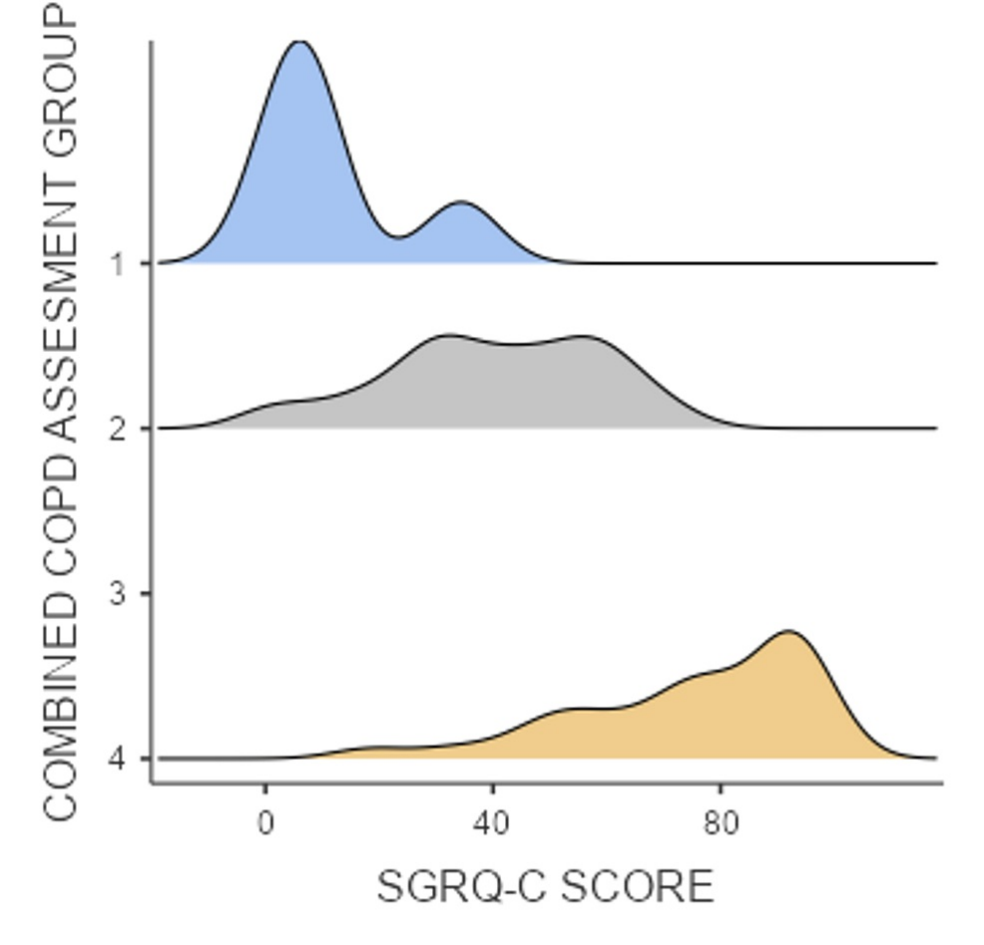
Density plot showing SGRQ-C score among various combined COPD GOLD assessment groups. GOLD group A: 1; GOLD group B: 2; GOLD group C: 3; and GOLD group D: 4. GOLD, Global Initiative for Chronic Obstructive Lung Disease; COPD, chronic obstructive pulmonary disease; SGRQ-C, St. George's Respiratory Questionnaire for chronic obstructive pulmonary disease.

There was a significant correlation between higher GOLD groups and higher SGRQ-C scores in 43 (58.5%) patients with depression with an average SGRQ-C score of 67.5 and 16 (21.9%) patients with moderate to severe anxiety with an average SGRQ-C score of 78.9. Average SGRQ-C scores for GOLD A, B, C, and D groups averaged 11.5, 40.2, 86.1, and 75.0, respectively, and the density plot is shown in Figure 2.

GOLD groups-based assessment of TOPD severity did not demonstrate a significant correlation with anxiety, depression, CRP level, WBC count, or serum fibrinogen. High serum fibrinogen levels were not correlated with high HAM-D scores, nor were serum fibrinogen levels and CRP levels correlated with high HAM-A scores.

## Discussion

There was a strong correlation between a history of TB and airflow obstruction among individuals aged ≥40 years with mental health issues in a population-based, single study of TOPD patients in a metropolitan city in southern India. The current authors believe that this is the first population-based study to explore this association with GOLD groups, TOPD, and inflammatory biomarkers in patients having anxiety and depression.

Although the exact mechanism of obstructive airways in post-TB patients is unclear, bronchiectasis, bronchiolar narrowing, bronchiolitis obliterans, and accelerated emphysematous changes may contribute to the obstruction of airflow due to TB. TB lesions may involve non-cartilaginous small airways with an internal diameter <2 mm, resulting in airflow obstruction. The presence of air trapping on a CT scan of the thorax associated with reduced attenuation on expiratory CT scans is also indicative of TB as a cause of obliterative bronchiolitis. Other lesions can appear radiographically as centrilobular nodules, "tree-in-bud"-like appearances, and poorly demarcated nodules. Despite the resolution of endobronchial and parenchymal changes, certain radiologic features may persist after TB treatment [17-19]. Among patients with a history of TB in developing countries, bronchiectasis is common, causing airflow obstructions and bronchial stenosis causing airflow limitation [20].

In bronchiectasis, endobronchial obstruction, peribronchial fibrosis, or enlarged lymph nodes are the main causes of chronic airflow obstruction [21]. A common sequela of scarred lung parenchyma due to TB is tractional bronchiectasis. During a two-year prospective study at Phramongkutklao Hospital in Bangkok, Thailand, it was found that of the 35 patients with bronchiectasis, 32% had TB, 24% had *Haemophilus influenzae*, 14% had *Klebsiella pneumoniae*, 6% had *Streptococcus pneumoniae*, and 6% had *Mycobacterium kansasii* and *Mycobacterium chelonae* [22]. An observational study of 53 patients over two years in western India found post-tuberculous sequelae bronchiectasis in 23% of patients [23]. In another study from Daegu, Korea, the most common CT findings in patients with tubercular destroyed lung tissue were atelectasis (84%) and emphysema with bronchiectasis (89%) [24].

As a result of serial changes in lung structure caused by pulmonary cavitation, airways may be obliterated or distorted, resulting in restricted airflow. Compared to patients without cavities, patients with cavities had significantly lower FEV_1_ at baseline and one month after starting TB treatment [25]. Based on a systematic review of 37 studies, the prevalence and pattern of post-TB lung disease was found to include cavitation (8.3-83.7%), bronchiectasis (35.0-86.0%), fibrosis (25.0-70.4%), nodules (25.0-55.8%), and emphysema (15.0-45.0%) [26].

A significant correlation between the HAM-D and HAM-A scores, as well as CRP levels, and WBC counts among patients who scored higher on the HAM-D was found in the current study, consistent with other studies showing inflammation to be a significant factor in the progression of both the disease and psychiatric comorbidity. A study conducted by Prnjavorac et al. found a significant relationship between CRP levels in the blood, fibrinogen levels, and the appearance of fibrosis on lung X-rays in patients with pulmonary TB. TB lung damage appears to be a result of multiple cell types and proteins orchestrating the development of lesions and progressing lesions. Alveolar macrophages are the first cells to become infected with *Mycobacterium tuberculosis* (MTB) in primary infection. Cavitation and caseation occur when these cells are activated, releasing inflammatory cytokines and chemokines that recruit both innate and adaptive immune cells to the infection site [27].

There are several ways in which neutrophils may damage tissue. Neutrophils are the most prevalent type of cells in the lungs infected with replicating MTB during active pulmonary TB, and cavitary lesions are primarily lined by neutrophils [28]. One study that analyzed lung biopsies revealed that the walls of the cavity stained positive for neutrophils expressing extracellular matrix-destructing matrix metalloproteinase (MMP)-8 and -9, causing calprotectin (S100A8/A9) to stimulate a large influx of destructive neutrophils and release chemokines (CXCL5) into the lung [29-31]. An increasing body of evidence suggests that neutrophil extracellular traps (MMP-8 and calprotectin) in TB may cause tissue destruction and pulmonary dysfunction like other lung diseases. The neutrophil extracellular trap is released upon activation and aims to capture and destroy bacteria. These traps contain chromatin fibers, histones, and proteases like myeloperoxidase (MPO), cathepsin G, and neutrophil-associated elastase, which may lead to severe pulmonary pathology [32-39].

The present study found that 43 (58.5%) of the 73 TOPD patients had some form of depression and 16 (21.9%) had moderate to severe anxiety symptoms. Patients with higher HAM-D scores also had higher CRP levels and WBC counts in addition to HAM-A scores, which is similar to other studies. Based on a systematic review (1981-2020), epidemiological evidence suggests an association between depression, anxiety, and TB. There is an important relationship between TB, anxiety, and depression in both high-income and low and middle-income countries, and countries with a high prevalence of the disease like India, Pakistan, and China have reported a strong association between the disease and mental disorders such as anxiety and depression. There is a higher incidence of depression and anxiety among patients with prolonged illness, and depression is more common in patients with pulmonary TB than in extrapulmonary TB. Depression can affect 1.71% to 87.5% of TB patients, and anxiety can affect 7.14% to 74% of TB patients. There is also a low to moderate rate of suicidal ideation (9%) and suicide attempts (3%) among TB patients [40-43].

It is well known that inflammation plays a significant role in both infectious and non-infectious diseases, and depression frequently occurs in conjunction with both infectious and non-infectious conditions [44]. MTB infection leads to several cytokines being produced [45]. The cellular, neural, and humoral pathways enable cytokines to reach the brain and overproduce within the parenchyma during inflammation. Proinflammatory cytokines such as interferon gamma (IFNγ), tumor necrosis factor-alpha (TNFα), interleukin 1 beta (IL-1β), and interleukin 6 (IL-6) can reach the brain parenchyma and play a role in the development of depressive disorder through regulating synaptic transmission, neuronal excitability, neuronal survival, and synaptic plasticity [46]. Inflammation induced by these mechanisms can cause depression by means of a variety of pathophysiological mechanisms, including disturbances in monoaminergic neurotransmission, oxidative injury, and impairment of hippocampal neuronal function [47,48].

There have been no studies that show that inflammatory biomarkers contribute to mortality and that psychiatric comorbidities such as anxiety and depression contribute to frequent exacerbations in TOPD patients. However, this has been studied in COPD patients. In a substudy of 1,673 participants in the SUMMIT trial with moderate COPD, fibrinogen and CRP were associated with an increased risk of death [49]. A study from the Hospital of Zunyi Medical University, Guizhou, China found that 63.5% of 307 patients with acute COPD exacerbations had depressive or anxious symptoms [50].

Compared with those without anxiety symptoms, COPD patients with comorbid anxiety disorders are more likely to report functional limitations, poorer exercise tolerance, and more acute exacerbations. When anxiety disorders are not adequately treated, they can become chronic, diminish self-esteem, predispose to suicidal ideation, and increase the chances of hospitalization. Depression and anxiety negatively affect the prognosis of COPD, increasing the risk of exacerbation and possibly death. On the other hand, COPD increases the risk of depression [51-53].

Several studies suggest that low-grade chronic inflammation might contribute to the association between depressive symptoms and pulmonary function. Chronic obstructive pulmonary disease and late-life depression are linked to increased inflammatory markers. A population-based study revealed that elevated levels of inflammatory biomarkers (IL-6 and CRP) contributed to the association of depressive symptoms with pulmonary obstruction [11,54-56]. TOPD patients are likely to have similar morbidities, progression, and complications.

The current study found TOPD patients in higher GOLD groups have a significant correlation between higher SGRQ-C scores in 58.5% of patients with depression, with an average SGRQ-C score of 67.5 and 21.9% patients with moderate to severe anxiety, with an average SGRQ-C score of 78.9. According to a study from Menoufia University in Egypt, quality of life has deteriorated among these patients, and it is associated with a significant increase in the SGRQ-C score, and it is crucial to perform psychological assessments and consult with a psychiatrist to improve symptoms, QOL, and address superimposed psychiatric symptoms [57]. Indicating that TOPD patients have poorer quality of life with additional psychiatric comorbidities.

Diseases such as TOPD, anxiety, and depression are prevalent in the population. These diseases are linked to substantial morbidity and mortality rates. They share some risk factors, especially systemic inflammation and that explains their syndemic relationship. Essentially, the presence of one favors the development of the other and worsens the disease. Patients with TOPD should be treated holistically in conjunction with those suffering from COPD, depression, and anxiety, as these conditions have significant co-morbidities. Hence, patient compliance and well-being can be negatively affected by poorly managed anxiety and depression.

Hence, timely and appropriate assessment of TOPD patients for co-morbidities like anxiety, depression, and inflammatory biomarkers should be done and appropriate interventions should be provided to improve the quality of life. Further studies are required regarding the identification of novel inflammatory biomarkers in the assessment of systemic inflammation, anxiety, and depression among TOPD patients.

The study has certain limitations. The sample size used is small. Before the development of obstructive airway disease (OAD), the amount of TB bacterial load and the extent of respiratory structure involvement were unknown. Additional associated risk factors that can contribute to the development of OADs have not been evaluated. Other risk factors that can contribute to anxiety and depression such as medical comorbidities, drug abuse, alcohol dependence, low educational attainment, poverty, social stigma, and social isolation have not been evaluated. Even though airflow obstruction in TB has received the most attention, mixed patterns of airflow obstruction and restrictive ventilatory defects have not been studied.

## Conclusions

Since the sample size of the present study is relatively small, TOPD prevalence in the general population is likely to be underestimated. Additional multicenter studies with a larger sample size are necessary to confirm the results. It is critical to remember that psychiatric comorbidity and obstructive pulmonary disease might develop after a diagnosis of pulmonary TB and that these issues must be addressed to improve the patients' quality of life. In view of variable results regarding inflammatory biomarkers influencing TOPD and psychiatric comorbidities, further research on specific inflammatory biomarkers could assist in diagnosing this condition in light of typical clinical and radiological features.

TOPD is relatively new as a clinical condition, so further studies are needed to assess its epidemiology and treatment options. Additionally, there is a lack of interventions to monitor cured pulmonary TB patients and treat post-tuberculous lung disease. Treatment of anxiety and depression symptoms effectively is critical for improving a patient's quality of life, optimizing healthcare utilization, and maximizing treatment outcomes.
